# Pheochromocytoma- and paraganglioma-triggered Takotsubo syndrome

**DOI:** 10.1007/s12020-019-02035-3

**Published:** 2019-08-09

**Authors:** Shams Y-Hassan, Henrik Falhammar

**Affiliations:** 1 Coronary Artery Disease Area, Heart and Vascular Theme, Karolinska Institutet and Karolinska University Hospital, Stockholm, Sweden; 2 Department of Endocrinology, Metabolism and Diabetes, Karolinska University Hospital, Stockholm, Sweden; 3 Departement of Molecular Medicine and Surgery, Karolinska Institutet, Stockholm, Sweden

**Keywords:** Takotsubo, Pheochromocytoma, Paraganglioma, Catecholamine, Broken heart, Myocardial stunning

## Abstract

Takotsubo syndrome (TS), also known as neurogenic stunned myocardium or broken heart syndrome, is a recognized acute cardiac syndrome. In about 70% of cases, the syndrome is preceded by an emotional or a physical stressor. Among the innumerable physical trigger factors that may induce TS are pheochromocytomas and paragangliomas (PPGLs). PPGL-associated cardiovascular complications as “myocarditis”, “myocardial infarction”, “reversible cardiomyopathies”, and “transient repolarization electrocardiographic changes” have been described since more than 70 years. During the last two decades, dozens of cases of PPGL-induced TS have been reported. PPGLs display increased catecholamine levels, sometimes massively elevated, which may trigger TS, most likely through hyperactivation of sympathetic nervous system including the cardiac sympathetic nerve terminal disruption with norepinephrine seethe and spillover. PPGL-induced TS is characterized by a dramatic clinical presentation with hemodynamic compromise and high complication rates. The prevalence of global and apical sparing pattern of TS in PPGL-induced TS is significantly higher than in other TS populations. In this report, the associations of PPGL-induced cardiovascular complications are analyzed, and clinical features, complications, outcome and treatment of PPGL-induced TS are reviewed.

## Introduction

Pheochromocytoma is a neuro-endocrine tumor arising from chromaffin cell in the adrenal medulla. Paraganglioma is also a neuro-endocrine tumor arising from extra-adrenal paraganglia [1]. The initial presentation of pheochromocytomas and paragangliomas (PPGLs) may be vague and symptoms and signs may be difficult to interpret [2]. Paroxysmal hypertension and the classic triad of headaches, diaphoresis, and palpitation were the symptoms of catecholamine excess that most commonly lead to the suspicion of PPGLs previously but are not so common presentation nowadays [1–4]. PPGLs are generally rare but may be more common in certain groups such as in patients with adrenal incidentalomas where 0.6–4.2% can be affected [5–7]. The majority of PPGLs are currently diagnosed during the work-up of an incidentaloma, then PPGL-related symptoms and lastly due to screening for a known hereditary syndrome (such as multiple endocrine neoplasia type 2, von Hippel Lindau syndrome, neurofibromatosis type 1, and mutations in succinate dehydrogenase B, C, and D) [2–4, 8]. Occasional cases are found with Cushing’s syndrome due to ectopic ACTH-production from the PPGL [9, 10], and all adrenal tumors should be investigated with a 1 mg overnight dexamethasone suppression test to exclude cortisol excess [11, 12]. In about three quarters of the patients with PPGLs, there will be overproduction of both epinephrine and norepinephrine. In the remainder, there is only norepinephrine secretion, especially in paragangliomas where the clear majority only has norepinephrine secretion [1, 8].

**Fig. 1:**
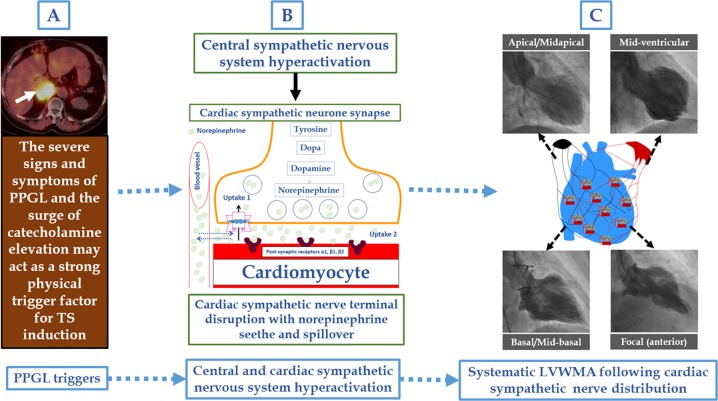
Pathogenesis of pheochromocytoma and paraganglioma (PPGL)-induced takotsubo syndrome (TS). PPGL is a strong physical trigger factor **a** and may trigger the autonomic (sympathetic) nervous system and result in sympathetic cardiac nerve terminal disruption and norepinephrine spillover **b**. This results in myocardial stunning, which most probably follow the cardiac sympathetic nerve distribution causing circumferential left ventricular wall motion abnormality (LVWMA) and may be localized to apical or mid-apical, midventricular, basal or rarely focal regions as illustrated in **c**

Takotsubo syndrome (TS), also known as neurogenic stunned myocardium or broken heart syndrome, is a recently recognized acute cardiac disease entity [13, 14]. The term takotsubo (tako = octopus, tsubo = a pot) was introduced in the beginning of 1990s by Sato and Dote to describe the left ventricular silhouette during systole in patients presenting with clinical features typical for that of myocardial infarction but demonstrated no obstructive coronary artery disease [15, 16]. The syndrome has a clinical presentation, electrocardiographic changes, and “myocardial infarction biomarker” elevation indistinguishable from that of an acute coronary syndrome (ACS) [14]. The main defining feature of TS is the regional and circumferential pattern of left ventricular wall motion abnormality (LVWMA) resulting in a conspicuous ballooning of the left ventricle during systole [17]. The left ventricular dysfunction extends beyond the coronary artery supply regions and is reversible with almost complete resolution of ventricular dysfunction in hours to weeks [13, 17]. The LVWMA may be localized to the apical, mid-apical, mid-ventricular, mid-basal or basal segments of the left ventricle (Fig. 1) [13]. A focal or global left ventricular contractile abnormality has also been reported [14, 18]. The right ventricle may be involved in about one third of the TS patients [19]. The syndrome is preceded by a trigger factor in about 70% of patients [14, 20]. Emotional triggers as death of a close relative or acute grief may trigger the syndrome and hence the term broken heart syndrome [21]. Innumerable physical triggers, extending from serious diseases as intracranial hemorrhages, sepsis to the most physiological processes as sexual intercourse, and pregnancy, may also trigger the syndrome [22]. One of the important and well-documented trigger factors is the external administration of epinephrine [23] or norepinephrine [24], and PPGLs [25, 26]. During the last two decades, dozens of cases of PPGL-triggered TS have been described [25, 26]. The aim of this review is to provide an up to date summary of available knowledge of PPGL-triggered TS.

## Pathogenesis

Severe signs and symptoms of PPGLs with massively increased catecholamine levels may act as a strong physical stressor triggering TS [14]. The most probable pathologic mechanism of PPGL-induced TS is illustrated in Fig. 1. In general, the pathogenesis of TS is still elusive and is discussed in detail elsewhere [13, 17]. In brief, several pathophysiological mechanisms for the development of TS have been proposed. The main proposed mechanisms are myocardial ischemia, left ventricular outlet tract obstruction (LVOTO), blood-borne catecholamine myocardial toxicity, epinephrine-induced switch in signal-trafficking, and autonomic nervous system dysfunction with sympathetic nervous system hyper-activation, including local cardiac sympathetic disruption and norepinephrine seethe and spillover. Evidences, challenging the first four proposed mechanisms and supporting the fifth one, are substantial and detailed elsewhere [13, 17]. The apical sparing patterns in mid-ventricular and basal types of TS strongly challenges the coronary myocardial ischemia, LVOTO, and the epinephrine-induced switch in signal trafficking hypotheses [17]. Long-lasting (hours or even days) ST-segment elevation in patients with TS and modest troponin elevation, the histopathological findings of contraction band necrosis, which is distinct from the myocardial infarction necrosis, also argue against coronary myocardial ischemia [13]. LVOTO is a complication rather than a cause of TS [17]. Apart from PPGLs, plasma epinephrine levels in TS is usually normal or moderately elevated, and the increased prevalence of apical sparing TS in epinephrine- or PPGL-induced TS also challenges a direct causal link between epinephrine and TS [27]. Worth mentioning is that myocardial ischemia, plasma catecholamines, and epinephrine administration may act as trigger factors, as any other physical stress factors, for TS induction [13, 28]. In addition, TS may cause myocardial infarction through left ventricular thrombus formation and coronary thromboembolism [29].

The evidences for the involvement of autonomic (sympathetic) nervous system including sympathetic cardiac nerve terminal disruption with norepinephrine seethe and spillover are substantial [17]. The deep anguish that arises from bereavement and induces TS in an individual argues for an excessive sympathetic stimulation of the myocardium likely mediated via the brain [30]. The TS-induction after acute intracranial diseases, such as intracranial and subarachnoid hemorrhage, thrombotic stroke, and epilepsy strongly suggests the involvement of the sympathetic nervous system in the pathophysiology of TS [13]. Signs of cardiac sympathetic denervation assessed by ^123^I-metaiodobenzylguanidine (^123^I-MIBG) scintigraphy have been demonstrated at regions with LVWMA in patients with TS. Elevated norepinephrine levels in the coronary sinus in patients with TS suggest increased local myocardial catecholamine release from the sympathetic cardiac nerve terminals [13]. Other evidences for the involvement of autonomic (sympathetic) nervous system including sympathetic cardiac nerve terminal disruption with norepinephrine seethe and spillover are delivered in detail in two reviews [13, 17].

## PPGL and cardiovascular complications including TS

Historically, during the last seven decades, pheochromocytoma has been described in association with “myocarditis”[31], “acute myocardial infarction” [32], “reversible cardiomyopathy “[33], “left ventricular dysfunction” [34], repolarization electrocardiographic changes, and different other cardiac complications as arrhythmias, cardiogenic shock, and pulmonary edema [34]. During the last two decades, dozens of cases of PPGL-triggered TS have been reported [25, 26], the majority being due to pheochromocytomas, however, paragangliomas were the culprit in 6%.

In addition to the characteristic circumferential LVWMA, TS has a clinical presentation identical to acute myocardial infarction, characteristic repolarization ECG changes, cardiac magnetic resonance (CMR) imaging findings consistent with focal myocarditis in one third of patients, and diverse cardiac complications as arrhythmias, pulmonary edema and cardiogenic shock. Having these TS features in mind, critical analysis of some of the reported PPGL-associated myocarditis, myocardial infarction, reversible cardiomyopathy and when enough investigations were performed reveals that those cases have features consistent with TS.

### PPGL and myocarditis

During more than 50 years and still cases of PPGL-induced acute “myocarditis”, commonly focal myocarditis, confirmed by either endomyocardial biopsy, autopsy [31], or by CMR imaging [35] have been reported. Critical analysis of at least some of these cases having been investigated with ECG and echocardiography shows that these patients also have features consistent with TS. As late as 2018, a 25-year-old man was reported with pheochromocytoma and CMR imaging findings consistent with “acute myocarditis” at the basal segments of the left ventricle [35]. However, the ECG findings of widespread ST-depression and the hypokinesis of the basal segments with both echocardiography and CMR imaging argue strongly for basal TS pattern (inverted TS) with late gadolinium enhancement at the basal segments. CMR imaging may show patchy late gadolinium enhancement in one third of patients with TS [36].

### PPGL and myocardial infarction

Cases of PPGL-induced chest pain and ECG findings of “myocardial infarction” have been reported [32, 37, 38]. Detailed analysis of some of those cases reveals findings consistent with TS. In 1983, McGonigle et al. [32] reported on a 38-year-old house wife with “recurrent myocardial infarction”. The patient was showed to have a pheochromocytoma. During one admission, the patient had reversible marked ST-elevation with tombstone appearance. The coronary arteries were normal. Left ventriculography revealed discrete left ventricular apical “aneurysm” with clot in the aneurysmal sac. With the current knowledge, this case can justifiably be deemed as recurrent TS triggered by a pheochromocytoma. Worth mentioning, tombstone ST-elevation has been described in TS [39]. In 1990, Nirgiotis et al. [38] described the association of “acute myocardial infarction” and pheochromocytoma in a 14-years-old girl. Coronary artery disease is very unlikely in this teenage girl and PPGL-triggered TS is in hindsight the most likely diagnosis. In 1993, Jessurun et al. [40] described the case of a 30-year-old pregnant woman that during the 33rd week of gestation suffering “acute anterior myocardial infarction with non-Q reinfarction” in association with pheochromocytoma. Echocardiography showed left ventricular dilatation, septal akinesis, and depressed left ventricular ejection fraction of 35%. She had normal coronary arteries. The description of this case is consistent with PPGL-triggered TS.

### PPGL and reversible cardiomyopathy

Cases suggested to have “reversible cardiomyopathy” induced by PPGL were reported 50 years ago [33]. The “suggested cardiomyopathy” in those patients improved by therapy with alpha receptor blockers prior to adrenalectomy [33]. Cases of reversible symmetrical deep T-wave inversion after treatment with phenoxybenzamine, a non-selective, irreversible alpha receptor blocker, in patients with “cardiac arrhythmia and cardiomyopathy” associated with pheochromocytoma have been described [41]. Cases of “reversible pheochromocytoma-induced cardiomyopathy”, which may have been global TS, have also been reported. In 1988, case records of the Massachusetts General Hospital [42] presented the case of a 20-year-old lady who was admitted because of the question of “dilated cardiomyopathy and stroke”. Investigation showed pheochromocytoma presented with “cardiomyopathy” complicated by embolic stroke of cardiac origin. Critical analysis of the echocardiography and left ventriculography reveals a typical case of reversible mid-basal (inverted) TS triggered by pheochromocytoma. In 2015, Batisse-Lignier et al. [43] reported on acute and chronic pheochromocytoma-induced cardiomyopathies. The authors systematically reviewed 145 published cases and classified them to “takotsubo cardiomyopathy” (49 patients) and “catecholamine cardiomyopathy” (96 patients). The cases were classified to TS if they were meeting the John Hopkins classifications criteria for TS [44]. Both groups had similar clinical presentation. Acute pulmonary edema was more frequent in “catecholamine cardiomyopathy”. Higher and better recovery of left ventricular ejection fraction was observed in patients with TS. The authors deemed that the two types of cardiomyopathies appeared to have different pathophysiological pathways. However, the two types of “cardiomyopathies” may be explained by the same pathophysiological mechanism and the difference is that the so called “catecholamine cardiomyopathy” is a more severe form of TS; the left ventricular wall motion abnormality is global and probably these patients had recurrent TS and a longer period of undiagnosed pheochromocytomas.

### PPGL and left ventricular dysfunction

Several other cases of reversible left ventricular dysfunction induced by PPGLs without mentioning TS have been described. In 1987, Shaw et al. [34] described the case of a 41-year-old man as a transient shock and myocardial impairment induced by a pheochromocytoma crisis. The patient had normal coronary arteries. The authors stated, “across sectional echocardiogram showed akinesia of all areas of the left ventricle except the basal segments”. Consequently, the findings in this case are typical for mid-apical TS pattern induced by PPGL. In 1989, Iga et al. [45] described a case entitled “reversible left ventricular wall motion impairment caused by pheochromocytoma”. A figure in the article shows an echocardiography finding typical for mid-apical ballooning consistent with mid-apical TS triggered by PPGL. In 2010, Roubille et al. [46] described the case of a 35-year-old woman with typical recurrent mid-basal (inverted) TS induced by pheochromocytoma but the cardiac condition was described as apical sparing mid and basal left ventricular dysfunction and the term TS was not mentioned.

### PPGL-induced hypercontracted sarcomere and contraction band necrosis

Contraction band necrosis (CBN), also referred to as coagulative myocytolysis and myofibrillar degeneration, is characterized histologically by dense irregular eosinophilic clumping of the sarcoplasm with intervening cleared areas [47]. One of the reported causes of CBN is both external administration of catecholamines and endogenous catecholamine elevations [47]. A consistent histopathological finding in patients with TS is the demonstration of CBN [48]. PPGL-induced hypercontracted sarcomere and CBN as that seen in TS has also been reported [49].

## Clinical features, complications, and outcomes of PPGL-induced TS

**Fig. 2:**
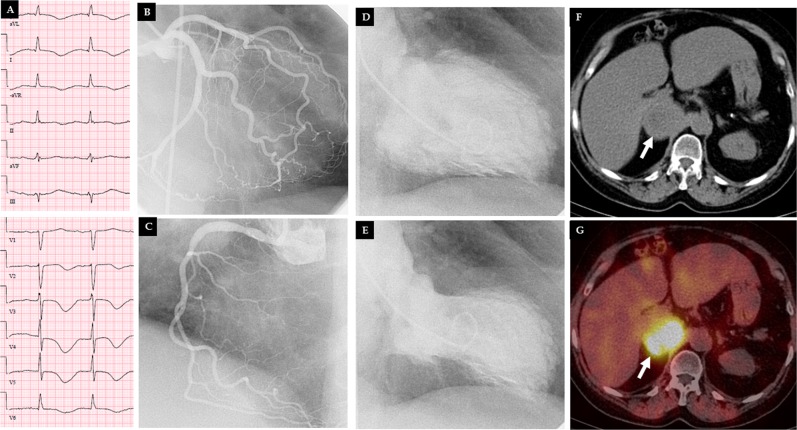
A typical case of mid-apical takotsubo syndrome (TS) pattern triggered by pheochromocytoma. The 12-lead electrocardiogram **a** shows T-wave inversions in the anterolateral leads with marked QTc prolongation 595 ms. Coronary angiography shows **b** normal left coronary artery (LCA), and **c** right coronary artery (RCA). Left ventriculography during diastole **d** and systole **e** shows mid-apical left ventricular ballooning consistent with TS. Abdominal computed tomography **g** reveals right adrenal tumor (white arrow); abdominal iodine-123 meta-iodobenzylguanidine (^123^I-MIBG) single-photon emission computed tomography displayed abnormal uptake of radiotracer within right adrenal pheochromocytoma (**h**, white arrow)

**Fig. 3:**
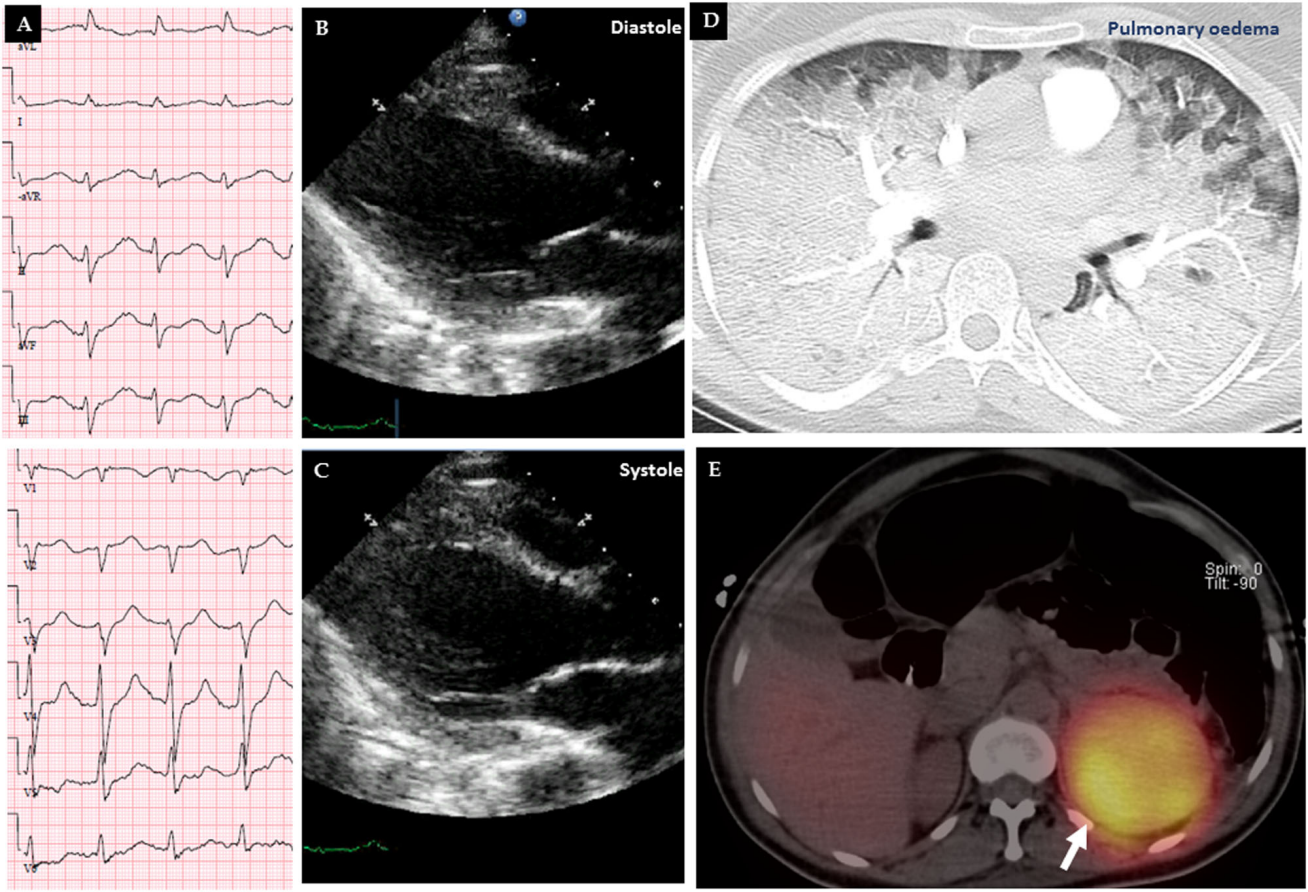
A case of mid-basal (inverted) takotsubo syndrome (TS) pattern triggered by pheochromocytoma. The 12-lead ECG reveals sinus tachycardia, ST-depressions in leads II, III, aVF, and V3–V6 **a**. Echocardiography during diastole **b** and systole **c** shows akinesia in the middle and basal segments of the left ventricle with normal contractions of the apical segments. Thoracic computed tomography scan with intravenous contrast showed signs suggestive of pulmonary edema **d** and the iodine-123 meta-iodobenzylguanidine (^123^I-MIBG) scan reveals a luminous left adrenal tumor mass due to high tracer uptake **e**

The reported mean age of the patient population in PPGL-induced TS was about 46 years, which was almost 20 years younger than all-triggered TS population [25, 50]. The prevalence of PPGL-induced TS in men was increased to 30% compared to only 10% in other all-TS populations [50]; however, women were still predominating in PPGL-induced TS. The most common presenting symptoms were chest and abdominal pain, dyspnea, and headache. Signs and symptoms suggestive of PPGL, such as pallor, profuse sweating, palpitations, labile blood pressure, and headache were present in almost 75% of patients [25]. The most common ECG changes during presentation were ST-elevation myocardial infarction (STEMI) like changes (38%), ST-depression (25%), T-wave inversion (14%), non-specific changes (9%), sinus tachycardia (11%), and tall peaked T-wave (1%) [25]. A characteristic feature of PPGL-induced TS was the increased prevalence of global TS pattern (20%) and basal pattern (30%) compared to 0% and 2.2% respectively in all-TS population [25, 50]. The apical sparing pattern of TS occurred in 35% (basal 30% and midventricular 5%) of patients with PPGL-induced TS [25, 51]. The “classical” apical (apical and mid-apical) TS pattern was significantly lower (44% compared to 83%) in PPGL-induced TS than all-TS population. Interestingly, the STEMI-like ECG changes and T-wave inversions were found almost exclusively in the apical or midventricular patterns of TS and the ST-depression or peaked T-wave occur in the basal patterns of TS [25]. The occurrence of the apical TS in less than half of the cases, apical sparing (basal and mid-ventricular) pattern in 35% of cases and the global pattern in 20% of cases strongly challenges the epinephrine-induced switch in the intracellular signal trafficking hypothesis causing TS proposed by Lyon et al. [52]. Figure 2 and 3 illustrate typical cases of mid-apical and mid-basal (inverted) TS patterns; both cases were triggered by pheochromocytomas.

It should be noted that the description of LVWMA is not always accurate in PPGL-induced TS. Patients with PPGL-induced TS may deteriorate rapidly, and the TS localization may transform from regional to global [53]. Such change has been well-demonstrated in the case reported by Flam et al. [53] where the patient had mid-basal TS pattern during the first admission day and this very rapidly progressed to severe biventricular failure during the following day. A similar case of paraganglioma-induced TS with development of biventricular heart failure was recently reported by Ferreira et al. [54]. Cases with PPGL-induced TS with such startling course complicated by respiratory failure, metabolic acidosis and cardiogenic shock have been reported [55, 56]. In a study of 140 patients with PPGLs, Giavarini et al. [57] found that 15 (11%) patients suffered “acute catecholamine cardiomyopathy”. Six out of 15 patients displayed classical mid-apical or inverted (mid-basal) TS. The remainder had severe extensive or global hypokinesia and a clinical manifestation of pulmonary edema. These findings may indicate that patients with PPGL-triggered global biventricular failure may in fact have global TS.

PPGL-induced TS is characterized by high complication rates. Two thirds of PPGL-induced TS developed some kinds of complications and almost one third of patients had multiple complications in a review study of 80 patients [25]. The complication rates in PPGL-induced TS are significantly higher than that in all-TS population reported by Templin et al. [50], Sharkey et al. [22], and Gianni et al. [58]. The combined endpoint of serious in-hospital complications, reported by Templin et al. [50] was 21.8%, which was significantly lower than the complication rates (68%) in PPGL-induced TS. The most important risk factors for the development of complications in PPGL-induced TS were; first, TS-localization pattern; the global TS localization had significantly higher hemodynamic complications than the apical TS-localization pattern. On the other hand, the thrombo-embolic complications occur mainly in the apical TS pattern [25]. Second, patients with PPGL-induced TS younger than 50 years suffered significantly higher complication rates compared to patients older than 50 years. Two out of 80 patients (2.5%) died [59, 60]; there was no difference in the in-hospital mortality between PPGL-induced TS and all-TS. Recurrence occurred in approximately a fifth of patients with PPGL-induced TS; the disease recurred twice in two cases and five times in one case [25, 61]. In one case, the patient developed multiple complications with multi-organ failure and resistant fever and died within hours during the recurrence, which occurred six months after the first attack [59]. The recurrence rate was significantly higher than that in all-TS reported by Templin et al. [50] of 3% and that reported by Sharkey et al. [22] of 5%. The high recurrence rate of TS in the PPGL-induced TS population is most probably attributed to the delay in the diagnosis of PPGLs where episodes of catecholamine excess have acted as a trigger factor [61]. The cardiac findings of PPGL-induced TS have been summarized in Table 1.

**Table 1:** Summary of cardiac findings in pheochromocytoma- and paraganglioma-triggered takotsubo syndrome

Presenting symptom: chest pain may occur in 42% of patients; a substantial number of patients may present with hemodynamic compromise and signs and symptoms suggestive of PPGL
Tachycardia with reported mean heart rate during presentation 116 ± 30/min
ECG changes: STEMI-like changes occur in more than one third of patients, ST depression in one fourth of patients, and T-wave inversion in 14%
Mild-moderate elevation of “myocardial infarction biomarkers” in 95% of patients
TS localization pattern is apical in 44%, midventricular in 5%, basal or inverted in 30%, global in 20, and focal in 1%. Left ventricular ejection fraction (LVEF) is markedly decreased (mean LVEF 27.7% ± 11.6) in most of the patients
High in-hospital complication rate, which may occur in two thirds of patients
Heart failure or pulmonary edema may occur in half of patients. Biventricular heart failure has also been reported
Cardiogenic shock occurs in one third of patients
Multiple complications (heart failure, pulmonary edema, cardiogenic shock, circulatory and respiratory failure) may occur in 31% of cases.
Cardiac thrombo-embolic complications may occur in 8% where most occur in apical pattern of TS
Arrhythmias may occur in 6.4%, cardiac arrest in 5% and electromechanical dissociation in 3.8%
Left ventricular outlet tract obstruction has also been reported
Relatively low in-hospital death rate (2.5%)
Recurrence rate occurs in 18% of patients and is usually due to delay of the PPGL diagnosis
PPGL-induced cardiomyopathy, myocarditis and myocardial infarction have been reported

*ECG* electrocardiogram, *PPGL* pheochromocytoma and paraganglioma, *TS* takotsubo syndrome, *STEMI* ST elevation myocardial infarction. For references, please see the main text

## Diagnosis of PPGL-induced TS

Cardiovascular symptoms as chest pain or dyspnea and sometimes abdominal pain associated with signs and symptoms of catecholamine excess, such as pallor, profuse sweating, palpitations, labile blood pressure, and headache should raise the suspicion of PPGL-induced TS [25]. New ECG changes and elevation of “myocardial infarction” biomarkers should lead to investigation with cardiac image study as echocardiography, which may display the typical LVWMA seen in TS [14]. Echocardiography is a feasible cardiac image study and can be repeated to follow the evolution of LVWMA, to detect TS-complications (as LVOTO, left ventricular thrombus, and development of biventricular heart failure), and to confirm the recovery of LVWMA [17]. CMR imaging is an excellent cardiac image study, which in addition to the above-mentioned findings seen in echocardiography [19], can also differentiate between myocardial infarction, acute myocarditis, and TS [14, 19, 20]. Because PPGL is an important, albeit rare, physical trigger factor for TS, checkup of catecholamine levels, preferably plasma free metanephrines or urinary fractionated metanephrines are essential [2, 8, 25, 62].

## Treatment of PPGL-induced TS

Treatment of TS is discussed elsewhere in details [17]. In brief, proper diagnosis and treatment of predisposing and triggering factors or diseases is crucial in the management of TS. Because of the transient nature of the disease, supportive therapy is indicated during the acute and subacute stages of the disease. In treatment of cardiogenic shock as a complication of PPGL-induced TS, it is fundamental to differentiate between LVOTO or primary pump failure as both conditions may cause severe hypotension and may be deemed as cardiogenic shock [17, 20]. The treatment of the two complications is quite different. Beta-blockers are crucial in the treatment of LVOTO. However, it should be noted that the use of beta-blockers is contraindicated in PPGLs in the absence of alpha-blockage due to unopposed stimulation of alpha-receptors and the potential risk of hypertensive crisis [62, 63]. Intravenous fluid administration may be considered in LVOTO caused by PPGL-induced TS [17, 20]. Extracorporeal life support as extracorporeal membrane oxygenation or left ventricular assist treatment as a bridge to recovery of left ventricular function is the most important measure in primary pump failure [17, 20]. Inotropic catecholamine administration is generally contraindicated in both LVOTO and primary pump failure [17], and in PPGL-induced LVOTO or primary pump failure it is likely even worse. Heart failure is treated with angiotensin converting enzyme inhibitor and beta-blockers with the caution mentioned above of unopposed stimulation of alpha-receptors. Patients with documented left ventricular thrombus, thrombo-embolic events and those with extensive mid-apical ballooning are treated with anticoagulation for 2–3 months or until the left ventricular dysfunction is resolved [29].

The definite treatment of PPGLs are the surgical removal of the tumor after confirming and localizing the tumor. Up titration of alpha–adrenergic antagonists are crucial in the perioperative period [8, 62]. Intravenous phentolamine, a reversible nonselective alpha-antagonist, and oral phenoxybenzamine, an irreversible nonselective alpha-antagonist, have traditionally been used for vasoconstrictive blockade and reduce the complications of malignant hypertension. Phenoxybenzamine use generally gives rise to orthostatic hypotension, reflex tachycardia, nasal congestion, dizziness and syncope. However, specific alpha_1_-antagonists, such as doxazosin, prazosin and terazosin, are also effective and often preferred for its shorter half-life, less adverse effects and less complicated management once the blood flow to the tumor has been ceased during surgery [62, 64]. Added benefits with specific alpha_1_-antagonists compared with phenoxybenzamine consist of avoidance of reflex tachycardia by the unopposed alpha_2_-receptor, i.e., preoperative beta-blockers is generally not necessary [64, 65]. However, beta-blockers are still necessary in the treatment of LVOTO (vide supra) in the acute stage. At least 1–2 weeks of alpha-blockage are needed before surgery, often a lot longer and with doses much higher than recommended in other conditions such as hypertension [8, 62, 64]. The timing of surgical removal in patients with PPGL-induced TS is usually later than in patients without PPGL-induced TS since the cardiac complications have to be controlled. Numerous echocardiogram may be needed before surgery to ascertain stable cardiac function. Realistically it usually takes at least 6 weeks, often months, before the patient with PPGL-induced TS can have surgery.

## Cardiac prognosis of PPGL-induced takotsubo syndrome

PPGL-induced TS is a serious disease with a dramatic clinical presentation and high complication rate (68%). However, the reported in-hospital mortality rate is relatively low (2.5%) [25]. The remainder recovers if treated appropriately [25]. The crucial points in improving the prognosis are as follows: first, to diagnose both conditions as early as possible. If PPGL continues to be undetected, there is a great risk of TS recurrence with additional risks of complications and permanent myocardial damage. The reported TS recurrence is high (18%) [25]. Second, two thirds of patients with PPGL-induced TS develop complications, more than one third develop cardiogenic shock, and in others progress to global biventricular heart failure [25, 53, 54]. The most important point is to avoid inotropic medications, which have a deleterious effect. Such patients should be treated with extracorporeal life support as extracorporeal membrane oxygenation. Di Vece et al. [66] have recently reported significantly lower in-hospital cardiac mortality in patients with TS complicated by cardiogenic shock if they were treated with cardiac mechanical support compared to those treated without.

## Conclusion

PPGL-triggered TS is characterized by a dramatic clinical presentation with high complication rates and may be fatal if not recognized and promptly managed. The prevalence of global and apical sparing pattern of TS in PPGL-induced TS is significantly higher than in other TS populations. PPGL-induced TS has often been misdiagnosed as “myocarditis”, “acute myocardial infarction”, and “reversible cardiomyopathy”. Patients with PPGL-induced TS may deteriorate rapidly and develop global and sometimes biventricular heart failure or cardiogenic shock. It is therefore essential for clinicians to be aware of the clinical presentation and manifestations of PPGL-induced TS since early identification can be life-saving.
